# Supplementary material for: Increased Hepatocyte Growth Factor Secretion by Placenta-Derived Mesenchymal Stem Cells Improves Ovarian Function in an Ovariectomized Rat Model via Vascular Remodeling by Wnt Signaling Activation
Source: Cells. 2023 Nov 25;12(23):2708. doi: 10.3390/cells12232708 (PMC10705748; doi:10.3390/cells12232708)
Supplement: Supplementary file 1 [file cells-12-02708-s001.zip › cells-2707072-supplementary.pdf]

Supplementary Table S1 Rat primer sequences using quantitative real time polymerase chain reaction.

Gene	Primer	Annealing Temperature (℃)	NM number
YAP	F: 5’-TTC GGC AGG CAA TAC GGA AT-3’<br>R: 5’-GTC ATC CCG GGA GAA GAC AC-3’	58	NM_001394328.1
EGFR	F: 5'-AGA TTG CAA AGG GCA TGA ACT AC-3'<br>R: 5'-ACA TTC CTG GCT GCC AAG TC-3'	58	NM_001393707.1
BMP15	F: 5'-ATC TGA TGT CCC TTG TCC TT-3'<br>R: 5'-CTC TGT ATG ATG GCA TGG TT-3'	54	NM_021670.1
IL-10	F: 5'-TGG CCC AGA AAT CAA GGA GC-3'<br>R: 5'-TTC TTC ACC TGC TCC ACT GC-3'	59	NM_012854.2
IL-6	F: 5'-ATC TGC CCT TCA GGA ACA GC-3'<br>R: 5'-AGC CTC CGA CTT GTG AAG TG-3'	59	NM_012589.2
GSK3β	F: 5'-GCT TCA ACC CCT TCA AAT GC-3'<br>R: 5'-GAC GCA GAA GCG GTG TTA TTG-3'	58	NM_032080.1
β-catenin	F: 5'-GTC TGA GGA CAA GCC ACA GGA CTA C-3'<br>R: 5'-AAT GTC CAG TCC GAG ATC AGC A-3'	62	NM_053357.2
GAPDH	F: 5'-TCC CTC AAG ATT GTC AGC AA-3'<br>R: 5'-AGA TCC ACA ACG GAT ACA TT-3'	55	NM_017008.4

Supplementary Table S2 Human primer sequences using quantitative real time polymerase chain reaction.

Gene	Primer	Annealing Temperature (℃)	NM number
hAlu	F: 5’-GGA GGC TGA GGC AGG AGA A-3’	60	NM_002715
	R: 5’-CGG AGT CTC GCT CTG TCG CCC A-3’		
EGFR	F: 5'-CCC TCC TGA GCT CTC TGA GT -3'	58	NM_001346897.2
	R: 5'-GTT TCC CCC TCT GGA GAT GC-3'		
BMP15	F: 5'-GGC TCC TAG GGC ATT CAC TG -3'	58	NM_005448.2
	R: 5'-GGT TGG GTT TTT CTG CAC CC-3'		
GAPDH	F: 5'-CTC CTC TTC GGC AGC ACA-3'	58	NM_001256799.3
	R: 5'-AAC GCT TCA CCT AAT TTG CGT-3'		

Supplementary Table S3 List of primary antibody used for Western blot analysis.

Gene	Catalog number	Company	Dilution rate
rabbit anti-GAPDH	LF-PA0018	Abfrontier	1:5000
rabbit anti-Lamin B1	ab16048	Abcam	1:1000
mouse anti-IL-6	ab9324	Abcam	1:1000
goat anti-TNFα	sc-1350	Santa cruz	1:100
rabbit anti-phospho c-Met	3077S	Cell signaling	1:1000
rabbit anti-c-Met	PA5-85951	Invitrogen	1:1000
rabbit anti-phospho LRP6	2568S	Cell signaling	1:1000
rabbit anti-LRP6	3395S	Cell signaling	1:1000
rabbit anti-phospho GSK3αβ	9331S	Cell signaling	1:1000
rabbit anti-GSK3αβ	5676S	Cell signaling	1:1000
rabbit anti-non-phospho (active) β-catenin	8814S	Cell signaling	1:1000
goat anti-Asef	sc-13275	Santa cruz	1:1000
mouse anti-Erg-1/2/3	sc-271048	Santa cruz	1:1000
rabbit anti-BMP15	MBS2516631	Mybiosource	1:1000
rabbit anti-EGF receptor	2232S	Cell signaling	1:1000
